# Study on the Extraction of Nervonic Acid from the Oil of *Xanthoceras sorbifolium* Bunge Seeds

**DOI:** 10.3390/foods13172757

**Published:** 2024-08-29

**Authors:** Hui Gao, Jie Sun, Xuan Guo, Ziyan Zhang, He Liu, Zhiran Zhang, Mengkai Liu, Sen Zhou, Shengxin Li, Tingting Zhang

**Affiliations:** College of Life Sciences, Qingdao University, Qingdao 266071, China; gaochuqi01@163.com (H.G.); sjj605@163.com (J.S.); heliu1997@126.com (H.L.);

**Keywords:** *Xanthoceras sorbifolium* Bunge seed oil, fatty acid, nervonic acid, thermogravimetry

## Abstract

Seven fatty acids were detected by GC-MS in *Xanthoceras sorbifolium* Bunge seed oil extracted at different temperatures, including Palmitic acid C16:0, Stearic acid C18:0, Oleic acid C18:1, Eicosenoic acid C20:1, Docosenoic acid C22:1, Tetracosenoic acid C24:1, and Linoleic acid C18:2. The highest content of nervonic acid (NA) was found in *Xanthoceras sorbifolium* Bunge seed oil extracted at 70 °C. Three methods were selected to analyze the extraction rate of nervonic acid in *Xanthoceras sorbifolium* Bunge seed oil, including urea complexation, low-temperature solvent crystallization, and a combined treatment using these two methods. The final content of nervonic acid obtained was 14.07%, 19.66%, and 40.17%, respectively. The combined treatment method increased the purity of nervonic acid in *Xanthoceras sorbifolium* Bunge seed oil by 12.62 times. Meanwhile, thermogravimetric behavior analysis of samples extracted using different methods was conducted by thermogravimetric analyzer, which suggested that the thermal stability of the samples extracted by the combined treatment was enhanced. These results can provide a new process parameter and scientific basis for the extraction of NA. At the same time, FTIR and NMR were also used to characterize the combined extraction sample, and the structure of the samples was proved.

## 1. Introduction

*Xanthoceras sorbifolium* Bunge, a native shrub commonly found in northern China, is a valuable woody oleiferous plant with significant economic importance [1,2]. This plant is utilized in the production of biofuels, smokeless lighting oils, and edible oils [3,4]. The oil content of the *Xanthoceras sorbifolium* Bunge seeds is 55–65% [5], and the oil exhibits a high unsaturated fatty acid content of more than 90% [6], predominantly composed of oleic acid and linoleic acid [7]. Additionally, the presence of the valuable monounsaturated fatty acid nervonic acid (NA) is noted, with a measured content of 1.83 ± 0.21% [8] in the oil. NA is a functional monounsaturated, very-long-chain fatty acid [9,10] (VLCFA) with a total of 24 carbons and only one double bond on the ninth carbon [11] (C24:1n-9). NA combines with sphingosine through amide bonds to form sphingolipid, which is an important component of white matter and myelinated nerve fiber [12,13]. NA can improve the function of brain, repair damage of central nervous system, and boost immunity. Intake of breast milk high in neuronic acid promotes the intellectual development of preemie [14,15,16]. The efficacy of NA has led to an increasing demand for it from consumers all over the world.

NA was first discovered in mammals and later isolated in shark oil, but in very small quantities and the process is not environmentally friendly. In addition, chemical and biological synthesis has also become a method of obtaining NA [17]. Two chemical synthesis routes of methyl erucic acid or oleic acid and suberate as the raw materials have been explored and used in obtaining NA [14]. However, these two chemical synthesis routes have the disadvantages of low productivity and lots of by-products, which make them unsuitable for large-scale production. NA is currently mainly derived from plants. Plants can be used for the extraction and purification of NA by obtaining the corresponding plant oil as material. The large number of structurally similar fatty acid species in plant oils makes it difficult to isolate and purify NA. Therefore, specific techniques are available for isolation and purification of NA, such as urea complexation, low temperature solvent crystallization, and other techniques.

Urea complexation is a classical method for separating saturated and unsaturated hydrocarbon chains. Saturated or monounsaturated fatty acids readily enter the space structure of the urea molecule and form stable crystal inclusions [12]. Fatty acids with branched carbon chains or many double bonds affect complex stability [18] or do not form complexes with the urea to achieve separation. Wang [19] et al. purified unsaturated fatty acid, α-linolenic acid, from perilla seed oil by utilizing the principle of urea complexation. Low-temperature solvent crystallization is an enrichment and purification method based on the difference in solubility and freezing point of mixed fatty acids in solvents at low temperature. Gao [20] et al. carried out the extraction of neuronic acid from *Acer truncatum* Bunge oil by using molecular distillation, urea complexation, and solvent crystallization. The urea complexation method can further increase the content of NA, but it cannot reduce the content of oleic acid and linoleic acid. However, the low-temperature solvent crystallization method can be effective solution to this problem. The combination of the two methods reduces the contents of oleic acid and linoleic acid, and further improves the purity of NA.

**Figure 1:**
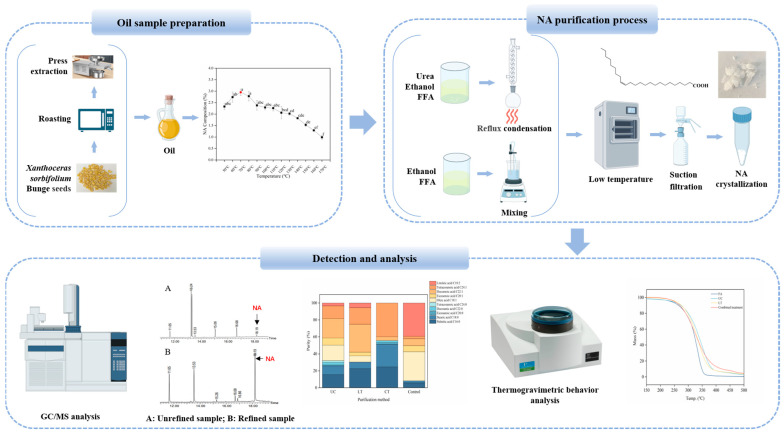
Flow chart of nervonic acid extraction. (A: Unrefined oil sample; B: Refined oil sample; NA is nervonic acid.)

NA has neuroprotective effects on nerve cells [21], the ability to improve memory [13], the effect of inhibiting the growth of tumor cells [22]. The health benefits of nervonic acid have garnered market attention. However, NA is relatively difficult to synthesize in the human body and is obtained through dietary sources. With the continuous advancement of separation and purification technologies, it has become a consensus to develop functional products related to NA in the field. The development of these products requires higher-content, process-appropriate natural sources of NA. Consequently, *Xanthoceras sorbifolium* Bunge seed oil has emerged as a viable raw material for the extraction of natural nervonic acid. Therefore, there were three methods, urea complexation, low-temperature solvent crystallization, and combined treatment, used for the extraction and purification of NA from the seed oil of *Xanthoceras sorbifolium* Bunge to obtain high-purity NA samples in this article. Meanwhile, thermogravimetric behavior was used to analyze the stability of the samples at different purities of NA. Figure 1 is the flow chart of the whole article. The extraction process involved in the article has the advantages of simple operation, environmentally friendly reagents, and a remarkable effect, which can provide a new process parameter and a scientific basis for the extraction of NA and expand the market application of *Xanthoceras sorbifolium* Bunge.

## 2. Materials and Methods

### 2.1. Materials and Chemicals

*Xanthoceras sorbifolium* Bunge seeds were provided by Shandong Woqi Agricultural Development Co., Ltd. (Weifang, China). The shell of the seed was removed, and the kernel remained intact. The dried seed kernels with moisture content of 10% were stored at 4 °C [7].

0.45 μm microfiltration membrane was purchased from Tianjin Jinteng experimental equipment Co., Ltd. (Tianjin, China). Sodium hydroxide (NaOH, AR), Ethanol absolute (AR), Sodiumsulfate (AR), Sulfuric acid (AR), Methanol (AR), n-Hexane (AR), and petroleum ether (boiling range 60–90 °C, AR) with analytical reagent (AR) were purchased from Sinopharm Chemical Reagent Co., Ltd. (Shanghai, China). Urea (AR) was purchased from Shanghai Aladdin Biochemical Technology Co., Ltd. (Shanghai, China).

### 2.2. Preparation of Seed Oil

The method for preparing *Xanthoceras sorbifolium* Bunge oil involves weighing 150 g of peeled *Xanthoceras sorbifolium* Bunge seeds and roasting them in an oven at 13 different temperatures (50–170 °C) for a duration of 25 min [7]. The roasted kernels were transferred to an automatic oil press for oil extraction. The obtained samples were centrifuged, and the supernatant was sealed in a glass vial at 4 °C and stored away from light.

### 2.3. Preparation of Free Fatty Acids

*Xanthoceras sorbifolium* Bunge seed oil was mixed with NaOH-methanol solution (1 mol/L) at a ratio of 1:6 (g/mL) and stirred at 85 °C, 40 rpm for 1–2 h. The pH was adjusted with 10% sulfuric acid to 2, and the solution was stirred in a water bath at 85 °C, 40 rpm for 1–2 h until the stratification of the solution was obvious. The upper layer is a clarified fatty acid layer, and the lower layer a glycerol-containing water layer. The upper layer was washed with distilled water until neutral, and anhydrous sodium sulfate was added to remove water.

### 2.4. Urea Complexation Method

Urea, methanol, and fatty acids were mixed and circulation reflux was performed at a ratio of 6:120:10 (g/mL/g) at 80 °C, 40 rpm for 20 min until the solution was clarified. It was then kept at −10 °C for 4 h. The solid samples were obtained by vacuum extraction. The solid sample was dissolved in distilled water to eliminate the urea and subsequently subjected to extraction using petroleum ether (boiling range 60–90 °C). After standing and stratification, the upper liquid was used to enrich the NA. NA was collected by removing petroleum ether with rotary evaporation at 60 °C and water with anhydrous sodium sulfate.

### 2.5. Low-Temperature Solvent Crystallization Method

FFA was dissolved in methanol solution at a ratio of 1:6 g/mL and kept at −20 °C and −60 °C for 4 h, respectively. The sample was quickly depressurization and filtration. The primary solid mixture sample was mixed with ethanol at a ratio of 1:3 g/mL and kept at −20 °C and −60 °C for 4 h, respectively. The second solid mixture sample was obtained.

### 2.6. Combined Treatment Method

Combined treatment is a method which combines the optimal process of urea complexation with the optimal process of low-temperature solvent crystallization. Refer to Section 2.4 and Section 2.5 for detailed operating procedures.

### 2.7. Fatty Acid Compositions Analysis

#### 2.7.1. Methylation of FFA

Methylation was performed according to the method reported by Mu [23] et al., with minor modifications. Free fatty acids in sample were methylated with the following method. First, a 50 mg sample was mixed with 2 mL 1% sulfuric acid-methanol and heated in a water bath at 70 °C for 30 min. Next, 2 mL n-hexane was added, the supernatant was taken and diluted 50 times, and then 0.22 μm microporous filter membrane was used for GC-MS analysis.

#### 2.7.2. Identification of Fatty Acid Composition

Fatty acid composition in the sample was determined by GC-MS (PerkinElmer, CLARUS SQ 8 GC/MS, Waltham, MA, USA). Chromatographic conditions were as follows: The chromatographic column was HP-5 capillary column (30 m × 250 µm, 0.25 µm, PerkinElmer, Waltham, MA, USA). The heating procedure was as follows: Initial temperature was 100 °C, the rate of 10 °C/min rose to 280 °C, and held for 10 min. Inlet temperature was 270 °C. The carrier gas was helium (purity ≥ 99.999%) at a flow rate of 2.0 mL/min. The solvent delay was 4 min. A split ratio was 10:1. The sample size was 1 µL.

For the mass spectrum condition, ionization was carried out in the electron ionization mode at 70 eV, and the resulting mass spectrum obtained was in the 30–550 *m*/*z* range. The MS source temperature was kept at 230 °C. NIST 17 library was used to qualitative analysis to determine the fatty acid composition. And peak area normalization method was used to quantitative analysis to determine the content of fatty acid.

### 2.8. Thermogravimetric

Thermogravimetric analysis was performed using a thermogravimetric analyzer (Perkin Elmer, STA8000, Waltham, MA, USA). Thermogravimetric behavior was carried out in nitrogen gas with a heating ramp of 10 °C min^−1^ and a temperature range of 50 °C to 500 °C.

### 2.9. FTIR Spectroscopy

A Nicolet infrared spectrometer (iS 50 FTIR; Nicolet, Glendale, WI, USA) was used to acquire FTIR spectra of *Xanthoceras sorbifolium* Bunge seed oil. A droplet of oil sample was applied onto the Platinum ATR at a temperature of 25 °C [24]. Wavenumber ranged from 4000 to 400 cm^−1^ for 16 scans [24,25].

### 2.10. NMR Spectroscopy

An amount of 50 mg of oil sample was dissolved in 600 μL of dimethyl sulfoxide (DMSO) and kept for 10 s [26]. NMR characterization was performed on Bruker Avance ARX 400 248 spectrometer (Billerica, MA, USA). ^1^H and ^13^C NMR was performed at operating frequency at 400 and 100 MHz, respectively [27].

### 2.11. Statistical Analysis

All samples tested were performed in triplicate and results were reported as mean ± standard deviation. Statistical analysis of data was performed using IBM SPSS 26 (SPSS Inc., Chicago, IL, USA) and Origin Pro 2018 (OriginLab, Northampton, MA, USA). Statistically significant differences (*p* < 0.05) in SPSS 26 software were used to perform one-way (ANOVA) on the data, and then Duncan’s multiple comparison test was performed.

## 3. Results and Discussion

### 3.1. Fatty Acid Composition and Content in Xanthoceras sorbifolium Bunge Seed Oil

As can be seen from the Table 1, seven fatty acids were detected in the *Xanthoceras sorbifolium* Bunge seed oil obtained by roasting treatment at different temperatures, including Palmitic acid C16:0, Stearic acid C18:0, Oleic acid C18:1, Eicosenoic acid C20:1, Docosenoic acid C22:1, Tetracosenoic acid C24:1, and Linoleic acid C18:2. There are two saturated fatty acids (28.57%), four monounsaturated fatty acids (71.43%), and one polyunsaturated fatty acid (14.29%), respectively. The fatty acid composition was consistent among the 12 samples and did not change with the temperature at which the oil was extracted. These results indicate that although the proportions of different fatty acid contents were changed in *Xanthoceras sorbifolium* Bunge seed oil extracted by roasting treatment at different temperatures, the main characteristics of the oil did not alter. This result is similar to that of Liu [28] et al., who detected changes in fatty acid content in olive oil at different temperatures. The unsaturated fatty acids, with above 87.07%, occupy the largest proportion in the oil; among these, oleic acid and linoleic acid are the main components of the unsaturated fatty acids in the seed oil, as well as being the two most abundant fatty acids in oil, with a content of about 31.48% to 39.19% and 35.42% to 39.33%, respectively. Linoleic acid is closely related to human life activities, involved in prostaglandin synthesis and other cell regeneration-related processes, and an essential polyunsaturated fatty acid for human beings [29]. Cellular lipid composition is altered by oleic acid with decreased the levels of polyunsaturated fatty acid acyl phospholipids and decreased the levels of ether-linked phospholipids [30]. Simultaneously, Oleic acid can reduce FAC-induced liver lipid peroxidation and damage in this way. However, the high content of oleic acid and linoleic acid in *Xanthoceras sorbifolium* Bunge seed oil has a negative effect on the purification of NA. Therefore, the removal of oleic acid and linoleic acid is one of the key points to obtain a higher purity of NA. In addition, the content of nervonic acid was significantly affected by temperature. The content of nervonic acid increases in the temperature range of 50–70 °C. However, at temperatures of 70–170 °C, the amount of nervonic acid decreases with increasing temperature. UFAs may undergo oxidation when exposed to high temperatures, and oxidation may occur near the ester carbonyl group preferentially [31]. UFAs oxidation is mainly carried out by an autocatalytic mechanism of free radicals induced by oxygen in the presence of initiators such as heat [32]. The content of nervonic acid with 2.95 ± 0.14% is the highest at 70 °C. Therefore, the subsequent purification process of nervonic acid is carried out on the basis of a 70 °C roasting treatment of pressed oil.

### 3.2. Urea Complexation

#### 3.2.1. Single Factor of Urea Complexation

The urea complexation method utilizes the spatial structure of the urea molecule to separate fatty acids with different chain lengths [33]. The amount of urea added can promote the complexation of fatty acids to some extent. However, polyunsaturated, monounsaturated, and saturated fatty acids have different encapsulation abilities with urea molecules due to their differences in spatial structure. Urea molecules forming a helix for every six molecules [14] spiral upward on the axis of the fatty acid. Consequently, a solid mixture composed of urea coated with fatty acid is formed. The formation of hexagonal crystals with urea is readily observed in saturated and monounsaturated fatty acids [14,34], which contrasts sharply with the behavior of PUFAs. Therefore, NA was extracted and purified based on this principle.

##### Effect of Ethanol Volume and Urea Mass on NA Content

**Figure 2:**
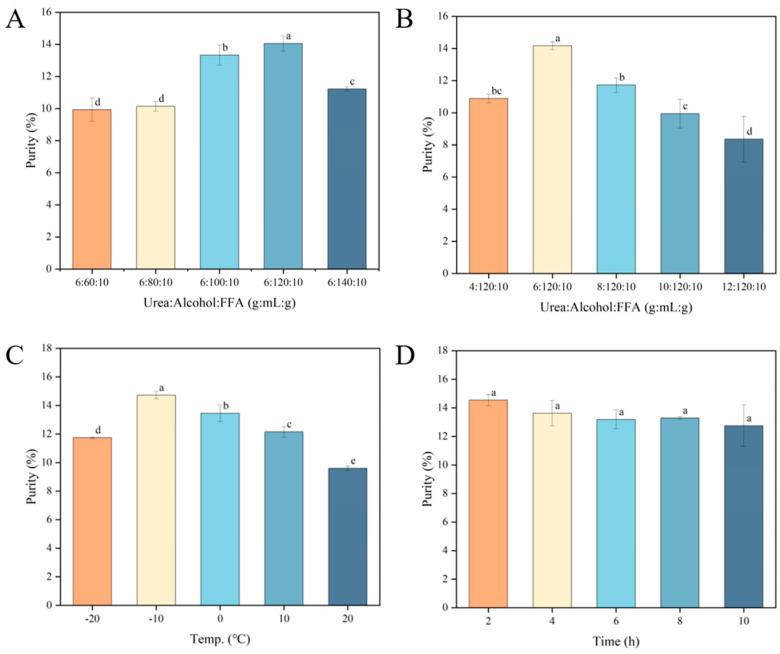
Single factor column chart of urea complexation. (**A**): Volume of ethanol is a variable; (**B**): Mass of urea is a variable; (**C**): Temperature is a variable; (**D**): Time is a variable. a–e indicated significant differences (*p* < 0.05).

The purity of NA was significantly influenced by the ethanol volume (*p* < 0.05). The addition of an optimal amount of ethanol will lead to a significant enhancement in the purity of NA. The highest purity of NA is observed at 6:120:10 (Figure 2A), with a value of 14.18 ± 0.25%. When the volume of ethanol is below 120 mL, the purity of NA decreases. This result is possibly due to the stronger binding ability of short-chain fatty acids to urea compared to NA. As this study centers around the essence of NA, it is deemed that the utmost purity of this compound (6:120:10) corresponds to the optimal ratio. It can be seen from Figure 2B that within a certain urea/fatty acid ratio, the purity of NA tends to increase and then decrease with increasing urea mass. This is similar to the results of María [35] et al., who extracted ultra-long-link fatty acids from MARINOL^®^ oil. Theoretically, the improvement of the mass of the urea is expected to enhance the purity of certain fatty acids, particularly saturated and monounsaturated, so that the formation of complexes with the urea is more readily observed [33]. However, the purity of polyunsaturated fatty acids significantly increased upon surpassing the urea mass threshold [19]. The reason for this may be that polyunsaturated fatty acids form complexes with excess urea, leading to an increase in purity. The increased purity of polyunsaturated fatty acids has a negative impact on the purity of NA [19].

The purity of NA exhibits marked variations with alterations in the alcohol/fat ratio. At the proportion of 6:120:10, the purity of NA reaches its zenith, thereby signifying the ideal composition for urea-inclusive NA.

##### Effect of Temperature and Time on NA Content

The complexation temperature is a critical parameter that significantly impacts the purity of NA. The purity of NA reached its peak at −10 °C (Figure 2C), with a value of 13.56 ± 1.68%. Urea complexation involves an exothermic process, and a suitably low temperature is advantageous for enhancing the concentration of unsaturated fatty acids in *Xanthoceras sorbifolium* Bunge seed oil [36]. However, urea precipitates rapidly at excessively low temperatures, resulting in monounsaturated fats not being complexed by urea [19]. This fact had a negative effect on the extraction of NA. Therefore, the optimal conditions for the enrichment of NA occur when the temperature is −10 °C.

The impact of the time of urea complexation on the purity of NA was found to be insignificant (*p* > 0.05), as evidenced by Figure 2D. However, the purity of NA shows a decreasing trend as the complexation time increases. The observed phenomenon can be attributed to the fact that short-chain fatty acids exhibit a higher propensity for forming complex solid mixture with urea compared to long-chain fatty acids as the complexation time increases.

#### 3.2.2. Statistical Analysis Using Response Surface Methodology

In order to find the optimal urea complexation condition, we used the response surface method to screen the optimal conditions for the experiment. Analysis of Variance (ANOVA) was performed using Design-Expert software 13. The independent variables included temperature, ethanol volume, and urea mass, as shown in Table 2. All parameters were selected based on their relevance in the urea-inclusion single-factor experiments.

Table 3 shows the results of model ANOVA for analyzing NA purity by response surface method. The results of variance analysis show the significance and fit of the model. R^2^ is 0.99, close to 1, indicating a good fit of experimental results. R_adj_^2^ and R_pre_^2^ are 0.98 and 0.89, respectively, and the difference between them is less than 0.2 [37] under reasonable conditions. Misfit 6.02 also shows that the results are not significant based on pure error. The above parameters prove that the model fits successfully. As can be seen from Table 3, A, B, C, AB, AC, BC, A^2^, B^2^, and C^3^ are all significant model terms, indicating that these factors (temperature, ethanol, urea, and interaction between the two of the three factors) are significant model terms [37]. Among them, temperature is the most significant factor (*p* = 0.0002), which significantly affects the purity of NA in the urea-inclusion experiments. Followed by ethanol volume and urea content, *p* values were 0.0012 and 0.0048, respectively.

Table 4 shows the small difference between the predicted values of the model and the values detected by the experiment. At the same time, Design-Expert 13 software was used to analyze the multiple regression model equation, and the prediction model equation was shown in the equation below.

(1) $$Y(\%)=-39.88-0.09A+0.62B+7.08C-0.00(A\times B)+0.04(A\times C)-0.02(B\times C)-0.01A^{2}-0.00B^{2}-0.40C^{2}$$

**Figure 3:**
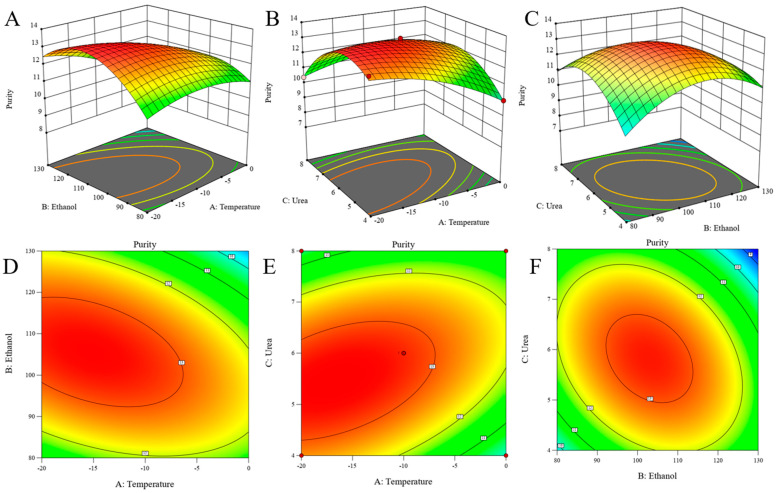
Interaction and contour plots of two-factor on urea complexation. (**A**): 3D plot of temperature vs. ethanol; (**B**): 3D plot of temperature vs. urea; (**C**): 3D plot of ethanol vs. urea; (**D**): Contour plots of temperature vs. ethanol; (**E**): Contour plots of temperature vs. urea; (**F**): Contour plots of ethanol vs. urea. Red indicates high content.

Three-dimensional (3D) response surface plots (Figure 3A–C) and contour plots (Figure 3D–F) reflect the interaction of temperature (°C) and ethanol volume (mL), temperature (°C) and urea mass (g), and ethanol volume (mL) and urea mass (g), respectively. The interactions between the three variables are shown in Figure 3. From Figure 3A–C, the 3D stereogram is steeper and the parabola opens downward; it was seen that there was a maximum value for the purity of NA. From the contour plots (Figure 3D–F), it can be seen that the contour plots were irregularly arranged with a regular ellipse in the center, and the response values varied significantly, indicating a significant interaction between the three factors [38]. When a single variable is held constant, the content of the NA will tend to increase and then decrease with the elevation of the corresponding interacting variable. NA purity has a maximum. The optimum reaction conditions were calculated by RSM numerical optimization method. The optimum conditions for the highest content of NA were temperature, −19.76 °C; ethanol volume, 111.27 mL; and urea mass, 5.42 g. After revision of the temperature to −20 °C, the ethanol volume was 111.27 mL and the urea mass was 5.42 g. The content of NA was 14.07%, and the predicted value was 13.45%, which indicated the experimental results were close to the theoretical results of the proposed model. This indicates that the model is effective in predicting the content of urea-coated NA [39].

### 3.3. Low-Temperature Solvent Crystallization

Fatty acids with difference solubility in solvents are mainly separated and purified by low-temperature solvent crystallization [20,40,41]. The solubility of short-chain unsaturated fatty acids and NA is quite different in a low-temperature solvent environment [20]. Low-temperature solvent crystallization aims to remove the influence of short-chain unsaturated fatty acids on the purity of NA. On the one hand, the content of oleic acid and linoleic acid was reduced significantly from 34.10 ± 0.16% and 39.33 ± 0.12% to 8.70 ± 0.19% and 4.31 ± 0.09%, respectively, by two times low-temperature solvent crystallization treatments at −20 °C. On the other hand, the purity of NA increased dramatically from 2.95 ± 0.14% to 18.34 ± 0.07%. Furthermore, the purity of NA was further improved to 19.66 ± 0.17% (Table 5) by two low-temperature solvent crystallization treatments at −60 °C. This can be attributed to the high solubility of short-chain unsaturated fatty acids in solvents, making them less prone to precipitation at low temperatures [42]. In contrast, saturated fatty acids and long chain monounsaturated fatty acids have low solubility in low-temperature solvents, making them more likely to form solid particles, thus making removal relatively easier [42]. Therefore, through the process of low-temperature crystallization, oleic acid and linoleic acid can be effectively eliminated, thereby further enhancing the purity of NA. The result is similar to that of Gao [20] et al., which showed that low-temperature crystallization was used to effectively remove C18~C20 unsaturated fatty acids.

### 3.4. Combined Treatment with Urea Complexation and Low-Temperature Solvent Crystallization

**Figure 4:**
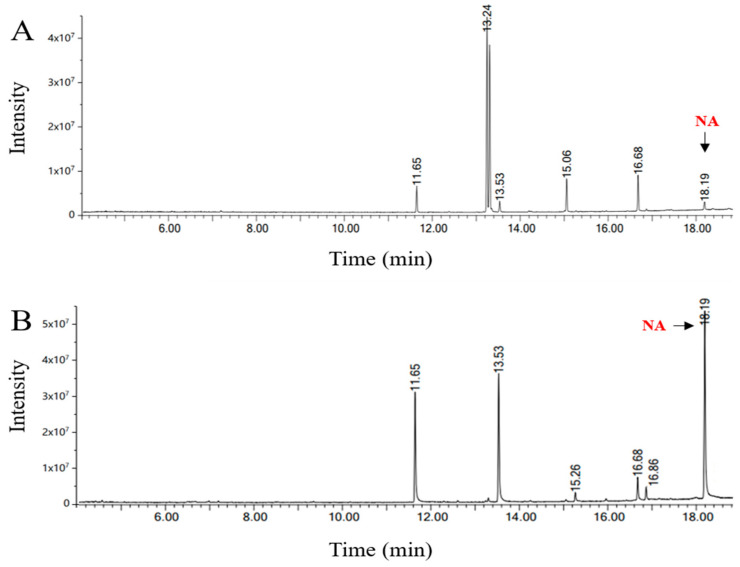
GC-MS chromatogram depicts the results of *Xanthoceras sorbifolium* Bunge seed oil of unrefined (as control (**A**)) and combined treatment (**B**). NA is nervonic acid.

**Figure 5:**
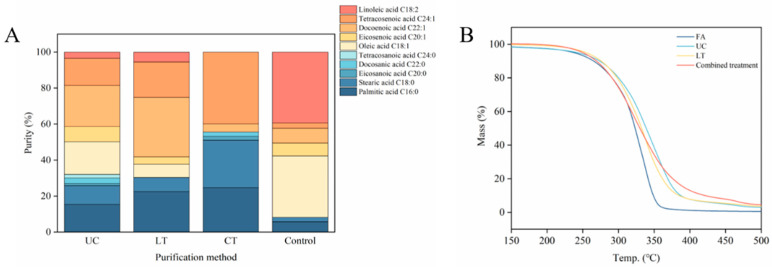
(**A**): Column stacking plot of fatty acid percentage for different purification methods. (**B**): Thermogravimetric analysis for different purification methods; UC: urea complexation method; LT: low-temperature solvent crystallization method; CT: combined treatment; Control: unpurified *Xanthoceras sorbifolium* Bunge seed oil.

Short-chain unsaturated fatty acids with the highest concentration are a pivotal factor impacting the purity of NA in *Xanthoceras sorbifolium* Bunge seed oil (Figure 4A). Both the single urea complexation method and the low-temperature solvent crystallization method can effectively eliminate short-chain unsaturated fatty acids. However, the combination of these two techniques entirely eradicates short-chain unsaturated fatty acids from the sample (Figure 4B). Remarkably, oleic acid and linoleic acid components were completely eliminated, resulting in a substantial improvement the purity of NA in the oil. As can be seen from Figure 5A, the main fatty acid components of sample experienced a shift, with NA emerging as the primary fatty acid by the combined purification process. The purity of NA was further increased from 2.95 ± 0.73% to 40.17 ± 0.91% (Table 6). This level of purity represents a remarkable 12.62-times increase compared to the control group consisting of unpurified fruit oil. Moreover, it has been validated that NA was extracted from the *Acer truncatum* Bunge oil by the urea complexation method and the low-temperature solvent crystallization method, and these methods effectively enhanced the purity of NA [20]. The yield of NA across the three methods is comparable (Table 6). Notably, the yield of the low-temperature solvent crystallization method is the lowest, likely due to temperature sensitivity during filtration. Although the yield of the combined treatment samples is not the highest, NA constitutes the largest proportion of the total fatty acid content. In summary, the combined treatment method has the best effect on the extraction of NA content among these three methods.

The process conditions of combined treatment can provide a novel technological reference and parameters and data basis for the extraction of NA and the utilization of *Xanthoceras sorbifolium* Bunge seed oil in various applications. At the same time, 55.61 ± 0.18% of saturated fatty acids remained in the sample. It is particularly important to find a way to effectively remove the saturated fatty acids in the subsequent experiments to improve the purity of the NA in the samples.

### 3.5. Thermogravimetric Behavior Analysis

The investigation of thermal stability in materials commonly involves the utilization of thermogravimetric analysis. The thermal stability of *Xanthoceras sorbifolium* Bunge seed oil samples with different extraction methods was analyzed using thermogravimetric curves in this experiment. Among these samples, the control group consists of unpurified *Xanthoceras sorbifolium* Bunge seed oil. Figure 5B illustrates variations in thermal stability among these samples attributed to the diverse extraction techniques employed and the combined treatment method. The thermogravimetric process is delineated into three distinct stages, including the first mass loss step (200~270 °C), the second mass loss step (270~450 °C peak temperature), and the final degradation step (450–500 °C). In all four samples analyzed, the initial phase of mass loss occurred within a temperature range of approximately 200 °C to 270 °C, signifying the onset of organic matter degradation within this interval. This degradation is attributed to the breakdown of unsaturated fatty acids at temperatures ranging from 200 °C to 230 °C [43].

The second mass loss step was observed in the temperature range of 270 °C to 450 °C. The decomposition rate of fatty acids in the sample gradually increases within this temperature range. Furthermore, the peak temperatures of carbonization decomposition for the four samples were 330 °C (Control), 350 °C (UC), 340 °C (LT), and 320 °C (CT), respectively. The difference in temperature range may be attributed to variations in the main fatty acid components and NA content within the samples. Notably, the combined treatment sample exhibited the lowest temperature, indicating a rapid carbonization rate possibly due to its high NA content. The thermal stability of the material is influenced by the degree of unsaturation present in its fatty acid chain [44]. Unsaturated fatty acids enhance material’s thermal sensitivity [43].

Within the temperature range of 450 °C to 500 °C, the samples undergo a final stage of degradation to achieve full carbonization. The temperatures necessary for complete carbonization are 360 °C (Control), 400 °C (UC), 400 °C (LT), and 430 °C (CT). It is worth noting that the co-treated sample requires a higher temperature for full carbonization compared to the other samples, attributed to its increased content of short-chain saturated fatty acids. This observation is consistent with prior research on *Terminalia catappa* fruit nut oils, which indicated that short-chain saturated fatty acids exhibit enhanced stability at elevated temperatures [43]. Overall, considering these three stages of mass loss collectively suggests that the stability of the combined treatment was enhanced.

### 3.6. FTIR Analysis

**Figure 6:**
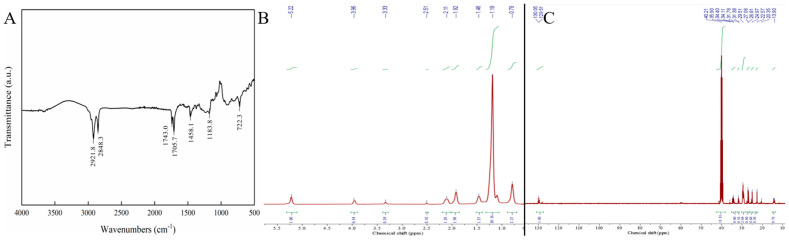
FTIR and NMR spectra of combined treated sample. (**A**): FTIR spectrum; (**B**): ^1^H−NMR spectrum; (**C**): ^13^C−NMR spectrum. The green line represents the peak area integral. The main line in the three graphs represents the peak of the spectrum.

FTIR spectroscopy has become a widely used technique in food research, particularly for the classification, adulteration detection, and authentication of edible oils and fats [24]. Triacylglycerides dominate the FTIR spectra of fats and oils due to their significant contribution to the peaks associated with fatty acids and triacylglyceride profiles [24,45]. Additionally, differences in the number of fatty acids within triacylglyceride molecules lead to shifts in the peaks, as the structure of the fatty acids influences the precise locations and shapes of these spectral features [45]. The FTIR spectrum of the combined treatment samples in the region of 4000–500 cm^−1^ are shown in Figure 6A. The peaks observed at 2921.8 cm^−^¹ and 2848.3 cm^−^¹ correspond to functional group hydrogen bond stretching. Specifically, they are indicative of C-H stretching vibrations found in saturated carbon and the symmetric stretching vibrations of the aliphatic CH_2_ groups present in triglycerides. Carbonyl stretching vibrations of esters in triacylglycerides give rise to the distinct peak at 1743 cm^−1^ and 1705.7 [24]. The peak observed at 1462 cm^−1^ is associated with the CH_2_ aliphatic bonds in triacylglycerides. There is a strong peak at 1163 cm^−1^, corresponding to the tensile vibration of the C- O ester group. The peak at 723 cm^−1^ was attributed to the vibration of the disubstituted olefins *cis*-HC = CH, which overlapped with the vibration of CH_2_. The results of the study are similar to the results of Hassan [24] et al.

### 3.7. NMR Analysis

#### 3.7.1. ^1^H-NMR Analysis

The NMR spectra of the combined treated sample are shown in Figure 6B,C. ^1^H-NMR of the CT sample (Figure 6B) reveals a wide peak at 11.79 ppm, which corresponds to the carboxyl protons of various fatty acid compounds. The peaks at 5.31 and 3.96 ppm are attributed to the olefin protons of unsaturated fatty acids. In the spectrum, the signal at 3.33 ppm is from solvent water, while the peak at 2.50 ppm corresponds to protons in the solvent DMSO. The high field range of 0.79–2.13 ppm displays several peaks associated with different saturated alkyl C-H protons of fatty acids. Specifically, the relatively high field signals at 2.12 and 1.92 ppm are attributed to alkyl protons near the carboxyl group, while the -CH_3_ proton signal appears at 0.79 ppm. The majority of remaining alkyl protons are represented by a strong signal at 1.19 ppm. Consequently, the NMR spectra provide reliable structural information confirming the presence of mixed fatty acids.

#### 3.7.2. ^13^C-NMR Analysis

^13^C-NMR of the CT compound reveals a peak at 174.87 ppm, attributed to the carboxyl carbons of various fatty acid compounds. Multiple peaks near 129.51 ppm correspond to the olefin carbon signals of UFAs [27]. The peak at 40.21 ppm represents the carbon signal from DMSO. In the high field range of 13–36 ppm, several peaks correspond to saturated alkyl carbon signals, reflecting the saturated carbons in different long-chain fatty acids. The signals at 34.40 and 31.45 ppm are attributed to alkyl carbons near the carboxyl and olefin groups, while the carbon signal for -CH_3_ appears at 13.90 ppm. A significant number of alkyl carbons are concentrated in the 20–30 ppm range, consistent with the characteristic features of fatty acid ^13^C-NMR. Thus, the NMR carbon spectrum confirms the structural information of the mixed fatty acids.

## 4. Conclusions

Seven fatty acids of *Xanthoceras sorbifolium* Bunge oil with different temperature treatments were detected by GC-MS in this study, including Palmitic acid C16:0, Stearic acid C18:0, Oleic acid C18:1, Eicosenoic acid C20:1, Docosenoic acid C22:1, Tetracosenoic acid C24:1, and Linoleic acid C18:2. Among fatty acid compositions, the highest purity of NA was found in the oil treated at 70 °C. There were three methods, urea complexation, low-temperature solvent crystallization, and a combined treatment using both urea complexation and low-temperature solvent crystallization, used to extract NA from *Xanthoceras sorbifolium* Bunge oil. The content of NA extracted using individual methods (urea complexation and low-temperature solvent crystallization) was 14.07% and 19.66%, respectively. However, after the combined treatment of urea complexation and low-temperature solvent crystallization, oleic acid and linoleic acid were completely removed, and the purity of nervonic acid was 40.17 ± 0.91%.

In addition, thermogravimetric analysis was also conducted using a thermogravimetric analyzer to analyze the thermal behavior of the samples obtained with three methods. The results suggested that the thermal stability of the samples was enhanced by the combined treatment method. The combined treatment not only improved the content of NA but also enhanced the thermal stability of the samples. Therefore, combined treatment method is the best extraction method of NA in *Xanthoceras sorbifolium* Bunge oil. At the same time, FTIR and NMR were used to characterize the combined treatment sample, and the structure of the combined treatment sample was confirmed.

## Figures and Tables

**Table 1:** Fatty acid composition (%) and content of *Xanthoceras sorbifolium* Bunge seed oil extracted by different temperatures.

Fatty Acids	Temperature (°C)												
	50 °C	60 °C	70 °C	80 °C	90 °C	100 °C	110 °C	120 °C	130 °C	140 °C	150 °C	160 °C	170 °C
**Saturated fatty acids**													
Palmitic acid C16:0	5.93 ± 0.87 ^ef^	8.26 ± 0.14 ^b^	5.78 ± 0.08 ^f^	8.97 ± 0.17 ^a^	8.29 ± 0.08 ^b^	6.23 ± 0.25 ^def^	5.98 ± 0.04 ^ef^	6.71 ± 0.28 ^cd^	6.46 ± 0.06 ^de^	6.70 ± 0.15 ^cd^	7.25 ± 0.08 ^c^	7.12 ± 0.11 ^c^	7.31 ± 0.10 ^c^
Stearic acid C18:0	3.94 ± 0.66 ^a^	2.8 ± 0.19 ^bc^	2.45 ± 0.12 ^c^	3.96 ± 0.08 ^a^	4.4 ± 0.62 ^a^	2.81 ± 0.11 ^bc^	2.89 ± 0.06 ^bc^	2.93 ± 0.06 ^bc^	2.93 ± 0.12 ^bc^	2.75 ± 0.14 ^bc^	3.18 ± 0.07 ^b^	2.89 ± 0.12 ^bc^	3.04 ± 0.11 ^bc^
**Monounsaturated fatty acids**													
Oleic acid C18:1	36.62 ± 1.25 ^b^	34.83 ± 0.55 ^c^	34.10 ± 0.16 ^cd^	32.74 ± 1.19 ^de^	31.48 ± 1.66 ^e^	37.09 ± 0.16 ^b^	34.16 ± 0.01 ^cd^	34.33 ± 0.31 ^c^	37.43 ± 0.07 ^b^	36.58 ± 0.12 ^b^	36.54 ± 0.09 ^b^	39.19 ± 0.11 ^a^	37.27 ± 0.18 ^b^
Eicosenoic acid C20:1	5.97 ± 0.14 ^e^	5.51 ± 0.15 ^f^	7.15 ± 0.07 ^cd^	6.99 ± 0.37 ^d^	7.51 ± 0.44 ^bc^	7.33 ± 0.11 ^cd^	7.79 ± 0.03 ^b^	7.49 ± 0.2 ^bc^	6.98 ± 0.12 ^d^	6.95 ± 0.14 ^d^	7.52 ± 0.11 ^bc^	5.88 ± 0.07 ^ef^	8.95 ± 0.14 ^a^
Docosenoic acid C22:1	7.07 ± 1.42 ^bc^	7.84 ± 0.74 ^bc^	8.23 ± 0.09 ^bc^	8.06 ± 1.04 ^bc^	10.52 ± 1.82 ^a^	8.23 ± 0.13 ^bc^	8.66 ± 0.07 ^b^	7.55 ± 0.39 ^bc^	7.11 ± 0.09 ^bc^	7.70 ± 0.11 ^bc^	6.52 ± 0.12 ^cd^	4.91 ± 0.10 ^e^	5.45 ± 0.11 ^de^
Tetracosenoic acid C24:1	2.33 ± 0.12 ^abc^	2.74 ± 0.22 ^ab^	2.95 ± 0.14 ^a^	2.78 ± 0.21 ^a^	2.38 ± 0.21 ^abc^	2.29 ± 0.11 ^abc^	2.26 ± 0.12 ^abc^	2.06 ± 0.27 ^bcd^	2.02 ± 0.07 ^cd^	1.82 ± 0.02 ^cde^	1.53 ± 0.07 ^def^	1.29 ± 0.04 ^ef^	0.99 ± 0.09 ^f^
**Polyunsaturated fatty acids**													
Linoleic acid C18:2	38.14 ± 0.76 ^bcde^	38.02 ± 0.28 ^cde^	39.33 ± 0.12 ^a^	36.5 ± 0.66 ^f^	35.42 ± 1.35 ^g^	37.09 ± 0.16 ^ef^	38.26 ± 0.19 ^abcd^	38.93 ± 0.2 ^abc^	37.43 ± 0.08 ^def^	36.58 ± 0.13 ^f^	36.54 ± 0.10 ^f^	39.19 ± 0.11 ^ab^	37.27 ± 0.09 ^def^
**Total**	7	7	7	7	7	7	7	7	7	7	7	7	7
**Saturated fatty acids (SFAs)**	9.87 ± 0.21	11.06 ± 0.33	8.23 ± 0.19	12.93 ± 0.25	12.69 ± 0.54	9.04 ± 0.26	8.87 ± 0.1	9.64 ± 0.34	9.38 ± 0.15	9.45 ± 0.02	10.43 ± 0.15	5.01 ± 0.10	10.35 ± 0.16
**Unsaturated fatty acids (UFAs)**	90.13 ± 0.21	88.94 ± 1.88	91.76 ± 0.30	87.07 ± 0.24	87.31 ± 0.54	92.02 ± 0.45	91.13 ± 0.36	90.36 ± 0.34	90.97 ± 0.07	89.63 ± 0.32	88.65 ± 0.22	90.47 ± 0.36	89.92 ± 0.32

^a–f^, values with different letters in a row indicate significant differences (*p* < 0.05).

**Table 2:** Coded and actual levels of the independent variables for the design of CCD experiment.

Independent Variables	Symbols	Coded Levels		
		−1	0	1
Temperature (°C)	A	−20	−10	0
Ethanol (mL)	B	80.00	105.00	130.00
Urea (g)	C	4.00	6.00	8.00

**Table 3:** ANOVA for the quadratic model of urea complexation.

Source	SS	df	MS	F-Value	*p*-Value	Remark
Model	39.16	9	4.35	92.62	<0.0001	significant
A	2.31	1	2.31	49.20	0.0002	
B	1.27	1	1.27	27.08	0.0012	
C	0.78	1	0.78	16.50	0.0048	
AB	2.54	1	2.54	54.16	0.0002	
AC	2.81	1	2.81	59.73	0.0001	
BC	3.69	1	3.69	78.48	<0.0001	
A^2^	1.40	1	1.40	29.89	0.0009	
B^2^	11.32	1	11.32	241.08	<0.0001	
C^2^	10.78	1	10.78	229.46	<0.0001	
Residual	0.33	7	0.05			
Lack of Fit	0.27	3	0.09	6.02	0.058	not significant
Pure Error	0.06	4	0.02			
Cor Total	39.48	16				

A: temperature (°C); B: ethanol volume (mL); C: urea mass (g); AB, AC, BC, A^2^, B^2^, C^2^ indicates the interaction between the two factors.

**Table 4:** Coded variables and experimental and predicted values for the production of hydrogen.

Run	Independent Variables			Purity (%)	
	A: Temperature (°C)	B: Ethanol (mL)	C: Urea (g)	Experimental	Predicted
1	−10	105	6	13.51	13.22
2	−10	130	8	8.91	8.31
3	−10	105	6	13.47	13.22
4	0	80	6	11.53	11.66
5	0	105	4	10.01	9.98
6	0	105	8	10.59	11.03
7	0	130	6	9.31	9.27
8	−20	130	6	11.77	11.94
9	−10	80	4	9.43	9.73
10	−10	80	8	11.10	11.03
11	−10	105	6	13.31	13.22
12	−10	105	6	13.42	13.22
13	−20	80	6	11.10	11.14
14	−10	105	6	13.38	13.22
15	−20	105	8	10.47	10.43
16	−10	130	4	10.78	10.85
17	−20	105	4	12.97	12.73

**Table 5:** Fatty acid composition after low-temperature solvent crystallization.

Fatty Acids	Composition (%)			
	−20 °C-One-Stage	−20 °C-Two-Stage	−60 °C-One-Stage	−60 °C-Two-Stage
**Saturated fatty acids**				
Palmitic acid C16:0	11.51 ± 0.11 ^c^	10.83 ± 0.09 ^d^	25.48 ± 0.13 ^a^	22.52 ± 0.08 ^b^
Stearic acid C18:0	4.94 ± 0.14 ^d^	22.86 ± 0.12 ^b^	23.64 ± 0.23 ^a^	7.85 ± 0.14 ^c^
**Monounsaturated fatty acids**				
Oleic acid C18:1	44.69 ± 0.05 ^a^	8.70 ± 0.19 ^c^	16.32 ± 0.11 ^b^	7.41 ± 0.17 ^d^
Eicosenoic acid C20:1	11.39 ± 0.09 ^a^	8.95 ± 0.21 ^b^	3.88 ± 0.16 ^c^	4.05 ± 0.28 ^c^
Docoenoic acid C22:1	13.75 ± 0.17 ^c^	26.01 ± 0.08 ^b^	11.95 ± 0.14 ^c^	33.04 ± 0.22 ^a^
Tetracosenoic acid C24:1	3.76 ± 0.06 ^d^	18.34 ± 0.07 ^b^	6.91 ± 0.15 ^c^	19.66 ± 0.17 ^a^
**Polyunsaturated fatty acids**				
Linoleic acid C18:2	9.96 ± 0.09 ^b^	4.31 ± 0.09 ^d^	11.82 ± 0.24 ^a^	5.47 ± 0.12 ^c^
**Total**	7	7	7	7
**Saturated fatty acids (SFAs)**	16.45 ± 0.03	33.69 ± 0.12	49.12 ± 0.26	30.37 ± 0.21
**Unsaturated fatty acids (UFAs)**	83.55 ± 0.23	66.31 ± 0.60	50.88 ± 0.55	69.63 ± 0.23

^a–d^: Different letters in a row indicate significant differences (*p* < 0.05).

**Table 6:** Fatty acid composition after combined treatment.

Fatty Acids	Composition (%)			RT (min)
	Urea Complexation	Low-Temperature Solvent Crystallization	Combined Treatment	
**Saturated fatty acids**				
Palmitic acid C16:0	15.43 ± 0.03 ^c^	22.52 ± 0.05 ^b^	24.66 ± 0.14 ^a^	11.65
Stearic acid C18:0	10.41 ± 0.09 ^b^	7.85 ± 0.18 ^c^	26.40 ± 0.18 ^a^	13.54
Eicosanoic acid C20:0	1.17 ± 0.10 ^b^	-	2.15 ± 0.08 ^a^	15.26
Docosanoic acid C22:0	3.05 ± 0.24 ^a^	-	2.40 ± 0.08 ^b^	16.87
Tetracosanoic acid C24:0	2.09 ± 0.24 ^a^	-	-	
**Monounsaturated fatty acids**				
Oleic acid C18:1	18.51 ± 0.15 ^a^	7.41 ± 0.15 ^b^	-	13.31
Eicosenoic acid C20:1	8.42 ± 0.12 ^a^	4.05 ± 0.15 ^b^	-	15.06
Docoenoic acid C22:1	22.93 ± 0.23 ^b^	33.04 ± 0.18 ^a^	4.22 ± 0.07 ^c^	16.70
Tetracosenoic acid C24:1	14.07 ± 0.10 ^c^	19.66 ± 0.06 ^b^	40.17 ± 0.23 ^a^	18.20
**Polyunsaturated fatty acids**				
Linoleic acid C18:2	3.92 ± 0.11 ^b^	5.47 ± 0.10 ^a^	-	13.25
**Total**	10	7	6	
**Saturated fatty acids (SFAs)**	32.15 ± 0.41	30.37 ± 0.21	55.61 ± 0.18	
**Unsaturated fatty acids (UFAs)**	67.85 ± 0.39	69.63 ± 0.01	44.39 ± 0.28	
**Yield (%)**	77.13	75.82	76.85	

RT: retention time; ^a–c^: Different letters in a row indicate significant differences (*p* < 0.05).

## Data Availability

The original contributions presented in the study are included in the article, further inquiries can be directed to the corresponding author.
